# Tribute to Professor Bongani Mawethu Mayosi: A friend, a brother, a mentor, a leader, a champion, a warrior and a true son of Africa

**Published:** 2018

**Authors:** Ogah Dr OS

**Affiliations:** Division of Cardiology, Department of Medicine, University College Hospital Ibadan, Ibadan, Oyo State, Nigeria

Since Friday 28 July 2018 when I got the news of the sudden death of Prof Bongani M Mayosi, I have been in ‘psychological shock’ and this will remain with me for a long time to come.

Bongani had called me a day or two earlier (while I was in Mombasa, Kenya, attending the annual conference of the Kenya Cardiac Society) to get an update on the INVICTUS trial in Nigeria. I had promised to call him back from Nigeria on Monday 30 July. It is therefore very hard for me to accept that a brother, a friend, a mentor, a warrior, a champion, a leader and a true son of Africa has suddenly left this side of eternity. This is devastating.

My first personal contact with Bongani was at the PASCAR renaissance meeting (4–9 February 2007) in Kenya (although he had earlier contacted some of us indirectly via e-mail through Prof Mpiko Ntsekhe/Dr Akinyemi Aje on 3 January 2007 to join the IMPI trial). At the PASCAR meeting, Bongani spoke with great passion and emotion and a very rare eloquence on the need for collaboration to tackle endemic diseases on the continent (especially ‘cardiovascular diseases of the poor’, such as rheumatic heart disease, tuberculous pericarditis and endemic cardiomyopathies).

Since then, Bongani has remained a source of leadership and inspiration, not only to me but also to my other colleagues from Nigeria, such as Prof Mahmoud Sani and Dr Dike Ojji. On 17 October 2007, I sent the proposal/protocol of the Nigerian Heart Failure Registry to him. Bongani replied and made vey useful comments but suggested we join the Continental HF registry (THESUS-HF).

Then started the era (contemporary) of very fruitful and rewarding collaboration in cardiovascular disease research through the instrumentality and able leadership of Prof Bongani Mayosi, Prof Karen Sliwa (who I first met at the WHO/ WELLCOME Trust workshop on secondary prevention of CVDs in LMIC in London, 6–8 June 2007) and Prof Albertino Damasceno (who I first met at the ISH hypertension teaching seminar in Maputo, Mozambique, 21–22 September 2006). These include the IMPI trial, REMEDY, RELY_AF registry, PAPUCO, BAHEF trial, CREOLE and INVICTUS trials.

For Bongani to leave us at a moment like this is very saddening and frustrating. We shall miss his useful advice. We shall miss his encouraging words and leadership.

‘O death, where is thy sting? O grave, where is thy victory?’

Our consolation is that you fought a good fight and ran your race well while you were on this side of eternity. The story of the African Union and United Nations Resolution on Rheumatic Heart Disease will not be complete without the name Bongani M Mayosi.

My deepest condolences go to his immediate family and all his associates worldwide. God willing, we shall keep the candle of collaboration he ignited burning.


                ‘Fading away like the stars of the morning,
Losing their light in the glorious sun –
Thus would we pass from the earth and its toiling,
Only remembered by what we have done.
Only remembered, only remembered,
Only remembered by what we have done.’

(Horatius Bonar)

Adieu Bongani Mayosi
